# Research on a Combined Harvester Grain Loss Detection Sensor Based on Vibration Characteristic Optimization

**DOI:** 10.3390/s25216740

**Published:** 2025-11-04

**Authors:** Guangyue Zhang, Tengxiang Yang, Man Chen, Jin Wang, Chengqian Jin

**Affiliations:** 1 Nanjing Institute for Agricultural Mechanization, Ministry of Agriculture and Rural Affairs, Nanjing 210014, China; zhangguangyue@caas.cn (G.Z.); yangtengxiang@caas.cn (T.Y.); chenman@caas.cn (M.C.); 82101232110@caas.cn (J.W.); 2 College of Agricultural Engineering and Food Science, Shandong University of Technology, Zibo 255049, China

**Keywords:** grain loss rate, sensing plate, modal analysis, combined harvester

## Abstract

This article aims to improve the real-time monitoring accuracy of the loss rate for grain combine harvesters by optimizing the sensor-sensitive plate structure, thereby addressing the problem of low detection efficiency in existing equipment. Based on Kirchhoff’s thin plate theory, COMSOL 6.0 software was utilized to conduct modal analysis and single-grain impact tests on rectangular and circular sensing plates fabricated from three materials: stainless steel, aluminum alloy, and cupronickel. The circular stainless steel sensing plate was identified as the optimal structure, whose natural frequency and sensitivity significantly outperform those of traditional rectangular plates. By integrating a signal processing strategy based on FFT (Fast Fourier Transform) spectrum analysis (band-pass filtering: 1.0~3.0 kHz, voltage threshold: 3.5 V) and a high-level duration counting algorithm, the system effectively distinguishes between grains and impurities and resolves the counting errors caused by multi-grain impacts and secondary rebounds. Field experiments demonstrate that the developed sensor exhibits strong anti-interference ability and high measurement accuracy, providing reliable technical support for reducing harvesting losses.

## 1. Introduction

The grain loss rate serves as a key indicator for evaluating the operational performance of combine harvesters, as well as an important basis for the real-time optimization and adjustment of working parameters. Thus, achieving accurate monitoring of the grain loss rate holds significant importance for the advancement of precision agriculture [1,2,3,4,5].

Currently, research on grain loss monitoring primarily centers on innovations in signal processing algorithms and monitoring principles [6,7,8,9,10], whereas studies concerning the structural optimization of the sensor’s core sensing component—the sensing plate itself—remain relatively limited. Nevertheless, the structural characteristics of the sensing plate directly dictate the fundamental performance of the sensor [11,12]. In compliance with the requirements specified in China’s national standards for the cleaning loss rate of soybean combine harvesters (not exceeding 3%), and based on calculations using typical soybean yield (approximately 250 kg per mu) and 1000-grain weight (approximately 250 g), the grain loss during the cleaning process amounts to approximately 11.25 g/s or 45 grains per second. Given the proportional relationship between grain loss in the monitoring area and total grain loss (with an average ratio of roughly 0.12) [13], it can be deduced that the actual detection frequency of the sensor should be higher than 6 grains per second.

In high-frequency collision scenarios, an excessively long decay time of the periodically damped oscillation signal generated by the sensing plate will lead to overlap and mutual interference between the collision signals of successive grains, thereby severely limiting the detection accuracy. Consequently, shortening the signal decay time and improving the system response speed constitute the core strategies for enhancing sensor performance. Notably, the decay time is directly determined by the vibration mode of the sensing plate, which is in turn jointly governed by its structural parameters, including material, shape, and thickness [14,15].

Based on this, this study aims to provide a design basis for sensor performance optimization by systematically investigating the influence of sensing plate structure on its dynamic characteristics. In this research, COMSOL software is employed to conduct modal analysis on sensing plates with different materials (e.g., 304 stainless steel, T6 aluminum alloy, and cupronickel plate), shapes, and thicknesses so as to explore their vibration behaviors. Furthermore, combined with single-grain collision tests, the signal response characteristics of sensing plates with various structures under actual working conditions are verified. Finally, the optimal combination of structural parameters for the sensing plate is determined, thereby improving the detection sensitivity and accuracy of the grain loss monitoring sensor.

## 2. Study on Vibration Characteristics of Sensing Plates with Different Structures

After grains collide with the sensing plate, the transient signal generated by the plate can be regarded as the rapidly damped vibration of the thin plate within its symmetry plane. Based on Kirchhoff’s thin plate small-deflection theory, the transverse vibration equation of an elastic thin plate can be established [16,17,18].

### 2.1. Transverse Vibration Equation of Thin Plates

#### 2.1.1. Transverse Vibration Equation of Rectangular Thin Plates

The differential equation for the vibration of a rectangular thin plate is as follows:

(1) $$\begin{array}{c} \nabla^{4}\omega \left(x,y,t\right)+\frac{\rho h}{D}\frac{\partial^{2}\omega }{\partial t^{2}}=0 \end{array}$$

When the boundary condition of the thin plate is fixed at four corners, the natural frequencies $f_{mn}$ of each order for the rectangular thin plate are given by:

(2) $$\begin{array}{c} f_{mn}=\frac{\pi }{2}\left[\frac{m^{2}}{a^{2}}+\frac{n^{2}}{b^{2}}\right]\sqrt{\frac{D}{\rho h}}\;\left(m,n=1,2\ldots \right) \end{array}$$

The deflection function is $W\left(x,y\right)$:

(3) $$\begin{array}{c} W_{mn}\left(x,y\right)=A_{mn}\cdot Sin\left(\frac{m\pi x}{a}\right)\cdot Sin\left(\frac{m\pi y}{b}\right)\;\left(m,n=1,2\ldots \right) \end{array}$$

In the formula: $\Delta^{2}=\frac{\partial^{2}\omega }{\partial x^{2}}+\frac{\partial^{2}\omega }{\partial y^{2}}\;$ is the Laplacian operator; $\;D=\frac{Eh}{12\left(1-\mu^{2}\right)}\;$ is the flexural rigidity, N/m; $A_{mn}\;$ is the coefficient of the deflection function W; E is the elastic modulus of the thin plate, GPa; μ is the Poisson’s ratio of the material; ρ is the density of the thin plate, kg/m^3^; h is the thickness of the thin plate, mm; a is the length of the thin plate, mm; b is the width of the thin plate, mm; m and n are the mode orders.

#### 2.1.2. Transverse Vibration Equation of a Circular Thin Plate

The vibration differential equation of a circular thin plate is:

(4) $$\begin{array}{c} \nabla^{4}\omega \left(r,\theta ,t\right)+\frac{\rho h}{D}\frac{\partial^{2}\omega }{\partial t^{2}}=0 \end{array}$$

When the boundary condition of the thin plate is fixed at four corners, the deflection function is $\;W\left(r,\theta \right)$:

(5) $$W_{m,n}(r,\theta )=J_{m}(k_{m,n}r)\cdot \left(C_{m}\cos(m\theta \right)+D_{m}\sin(m\theta ))$$

The natural frequencies of each order for a circular thin plate are ${\;f}_{mn}$:

(6) $$\begin{array}{c} f_{mn}=\frac{{\lambda^{2}}_{mn}}{4\pi a^{2}}\sqrt{\frac{Eh^{2}}{3\left(1-\mu^{2}\right)\rho }}\left(m=0,1,2\ldots n=1,2,3\ldots \right) \end{array}$$

(7) $$\begin{array}{c} \lambda_{m,n}=aK_{m,n} \end{array}$$

In the formula: $\nabla^{4}=(\frac{\partial^{2}}{\partial r^{2}}+\frac{1}{r}\frac{\partial }{\partial r}+\frac{1}{r^{2}}\frac{\partial^{2}}{\partial \theta^{2}}{)}^{2}\;$ is the polar coordinate biharmonic operator; $\;D=\frac{Eh^{3}}{12\left(1-\mu^{2}\right)}\;$ is the flexural rigidity, N/m; E is the elastic modulus of the thin plate, GPa; μ  is the Poisson’s ratio of the material; ρ  is the density of the thin plate, kg/m^3^; h is the thickness of the thin plate, mm; k is the frequency coefficient; a is the radius of the circular thin plate; $K_{m,n}\;$ is the “characteristic wave number” corresponding to modes m and n; $C_{m}$,$\;D_{m}$ is constant determined by the initial conditions of vibration.

The two core performance indicators of the grain loss monitoring sensor-output signal amplitude and signal decay time—are both directly determined by the vibration characteristics of the sensitive plate [11,19]. Firstly, the higher the first-order natural frequency of the sensitive plate, the faster the decay of its transient response. This allows the system to reach a steady state more quickly, thereby enabling the sensor to achieve a higher detection frequency and detect a greater number of grains per unit time. Secondly, the larger the deflection (deformation) of the sensitive plate at the collision point, the higher the amplitude of the piezoelectric signal (voltage) usually generated—this indicates a higher sensitivity of the sensor.

From the above vibration equation, it can be known that when parameters such as the elastic modulus *E*, thin plate density ρ, and Poisson’s ratio μ of the sensitive plate are determined, the natural frequency and deflection are mainly closely related to the structure of the sensitive plate. Therefore, selecting an appropriate sensitive plate structure is the key to improving the detection performance of the grain loss monitoring sensor. Based on this theory, this study will conduct in-depth analysis and optimization of sensitive plates with different structures and materials.

### 2.2. Differential Amplification Detection Method

**Figure 1:**
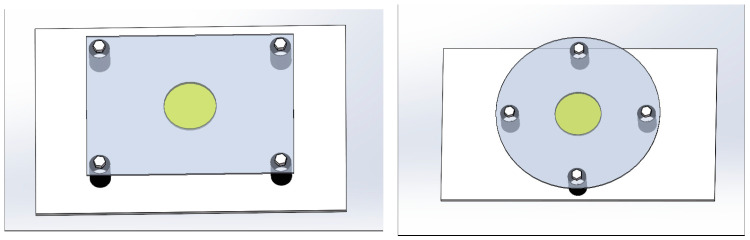
Sensor installation schematic.

**Figure 2:**
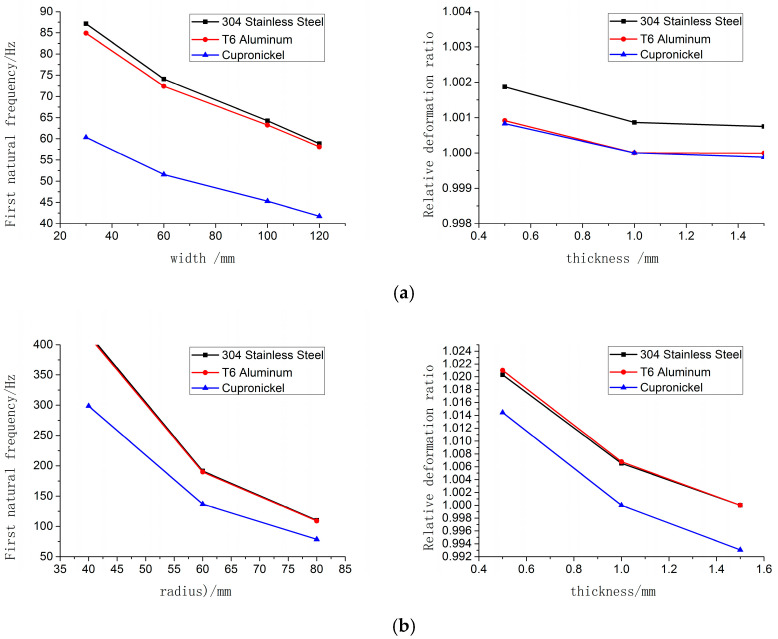
First order natural frequency and relative deformation rate versus variation curves of sensitive plates with different materials and shapes. (**a**) Curves of first-order natural frequency and relative deformation rate versus radius and thickness variations for circular sensitive plates of different materials. (**b**) Curves of first-order natural frequency and relative deformation rate versus radius and thick-ness variations for circular sensitive plates of different materials.

To compare the differences in dynamic performance between circular and rectangular sensing plates, this study conducted the following experimental design and analysis:

(1) Experimental Sample Design: Three typical metal materials—304 stainless steel, T6 aluminum alloy plate, and cupronickel plate—were selected as the sensing plates. Samples with combinations of different sizes and thicknesses were designed: the radius of the circular plates ranges from 40 mm to 80 mm; the rectangular plates have dimensions of 160 mm × (30–120 mm); and both types of sensing plates (circular and rectangular) were set with three thickness gradients, i.e., 0.5 mm, 1.0 mm, and 1.5 mm.
(2) Installation and Constraint Conditions: The boundary conditions of the sensitive plate are all set as the four-corner fixed mode. Piezoelectric elements were accurately bonded to the geometric center of the sensing plates using epoxy adhesive. To isolate external vibration interference, nitrile rubber shock-absorbing pads were installed between the sensing plates and the brackets. M4 stainless steel bolts were used as connecting fasteners, and spring washers were matched between the bolts and the plate bodies to prevent loosening caused by vibration, thus ensuring installation stability. The specific installation method is shown in Figure 1.

(3) Analysis Method and Result Presentation: Modal analysis was performed on each combined sample using COMSOL Multiphysics simulation software. The first-order natural frequency and relative deformation rate were extracted, and their variation curves with size and thickness were plotted (as shown in Figure 2). Through systematic analysis of the simulation results, the main conclusions are drawn as follows:

(1) Influence of Materials: Under the same size, cupronickel plates exhibit the lowest first-order natural frequency. For rectangular plates, the first-order natural frequency of 304 stainless steel plates is marginally higher than that of T6 aluminum plates; whereas for circular plates, the first-order natural frequency values of these two materials are relatively close to each other.
(2) Analysis of Sensitivity Potential: Under the same thickness condition, rectangular 304 stainless steel plates exhibit the largest relative deformation rate; this characteristic indicates that they may produce a higher signal amplitude. In contrast, the relative deformation rates of rectangular T6 aluminum plates and cupronickel plates are relatively lower. For circular plates, cupronickel plates show the smallest relative deformation rate, while the relative deformation rates of 304 stainless steel plates and T6 aluminum plates are relatively comparable.
(3) Shape Effect: Most importantly, under identical material and boundary conditions, the first-order natural frequency of the circular sensitive plate is significantly higher than that of the rectangular sensitive plate, with its relative deformation rate also being larger. This indicates that the circular plate structure holds advantages in two key performance indicators: response speed (corresponding to high natural frequency) and sensitivity (corresponding to large deformation rate). Its geometric symmetry and constraint configuration contribute to higher stiffness and superior vibration behaviors [20,21,22].

### 2.3. Verification of Vibration Characteristics of Sensitive Plates

**Figure 3:**
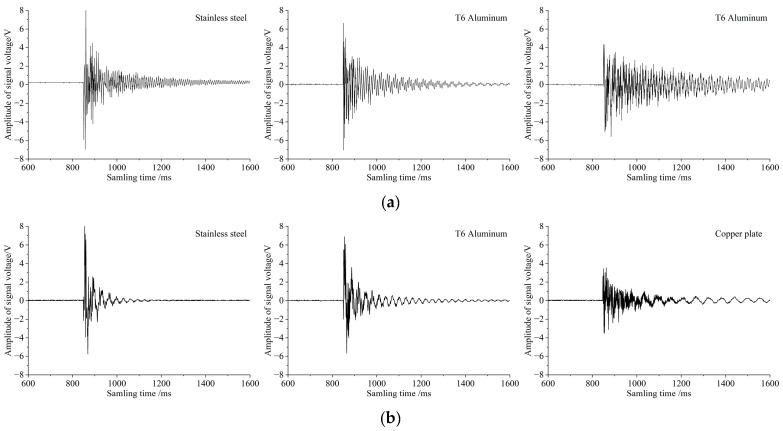
Voltage signal waveforms when grains collide with 1 mm-thick sensitive plates of different materials and shapes. (**a**) Voltage signal waveforms of rectangular sensitive plates made of different materials. (**b**) Voltage signal waves of circular sensitive plates made of different materials.

To validate the accuracy of the modal analysis results, a single-grain collision test platform was established. With piezoelectric ceramic sheets as sensitive elements, sensor specimens of different materials were fabricated, including rectangular plates (160 mm × 90 mm × 1 mm) and circular plates (radius (R) = 60 mm, thickness = 1 mm). Sensor signals were processed by a conditioning circuit (comprising a charge amplifier, a filter, and a voltage comparator) and then recorded using an oscilloscope. During testing, a single soybean grain (consistent across tests) was released to fall freely from a height of 20 cm directly above the center of the sensitive plate, ensuring consistency in collision position and excitation energy. The output signal waveforms are presented in Figure 3.

Analysis of the test results demonstrated a high degree of consistency with the simulation results:

(1) For rectangular plates: The 304 stainless steel plate exhibited the highest first-order natural frequency and the shortest signal decay time, while the cupronickel plate showed the lowest first-order natural frequency and a significantly longer signal decay time. Regarding voltage amplitude, the 304 stainless steel plate produced the highest output—attributed to its maximum relative deformation rate—followed by the T6 aluminum plate and the cupronickel plate in sequence.
(2) For circular plates: The 304 stainless steel plate and T6 aluminum plate exhibited comparable first-order natural frequencies and relative deformation rates, which in turn led to negligible differences in their signal decay times and voltage amplitudes. In contrast, the cupronickel plate was markedly inferior to the other two in overall performance. These results fully validate the core conclusion that “the first-order natural frequency is the primary factor affecting signal decay time, and the relative deformation rate is the main factor influencing voltage amplitude”.

**Figure 4:**
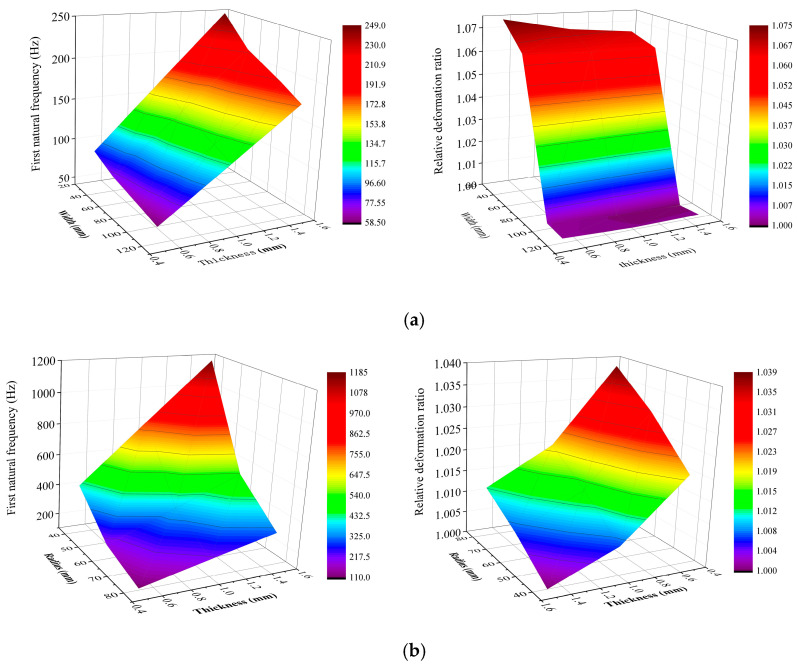
Relationship between first-order natural frequency, relative deformation rate and sensitive plate dimension. (**a**) Relationship between first-order natural frequency, relative deformation rate and width, thickness of stainless steel rectangular sensitive plates. (**b**) Relationship between first-order natural frequency, relative deformation rate and radius, thickness of stainless steel circular sensitive plates.

After comprehensively considering detection frequency, sensitivity, and material wear resistance (hardness), 304 stainless steel was determined to be the optimal material for the sensitive plate. Based on this conclusion, Figure 4 further demonstrates the relationship between the structural parameters and performance of the 304 stainless steel sensitive plate, from which the following rules are derived:

For rectangular plates: When the thickness exceeded 0.6 mm, the first-order natural frequency increased significantly, while the relative deformation rate decreased moderately. When the width exceeded 60 mm, the first-order natural frequency decline slowed down, whereas the relative deformation rate decreased noticeably. The parameter range yielding optimal performance was determined as width = 40–80 mm, thickness = 0.6–1.2 mm.

For circular plates: When the thickness exceeded 1 mm, the first-order natural frequency increased, while the relative deformation rate decreased significantly. When the radius exceeded 60 mm, the first-order natural frequency decreased, whereas the relative deformation rate increased. The parameter range yielding optimal performance was determined as radius = 40–70 mm, thickness = 0.5–1.0 mm.

In summary, this study validated the accuracy of the simulation model through experiments and finally identified the preferred material (304 stainless steel) and optimal structural parameter range (for both rectangular and circular plates) for the sensitive plate of grain loss sensors. This work lays a key foundation for the design and development of high-performance grain loss sensors.

## 3. Performance Tests and Result Analysis

### Analysis of Single-Grain Collision Experiment

**Figure 5:**
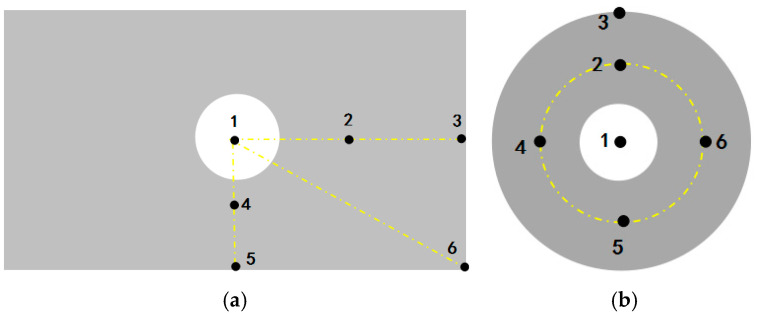
Measuring point distribution diagram of sensitive plates: (**a**) Measuring point distribution diagram of rectangular sensitive plates; (**b**) Measuring point distribution diagram of circular sensitive plates.

**Figure 6:**
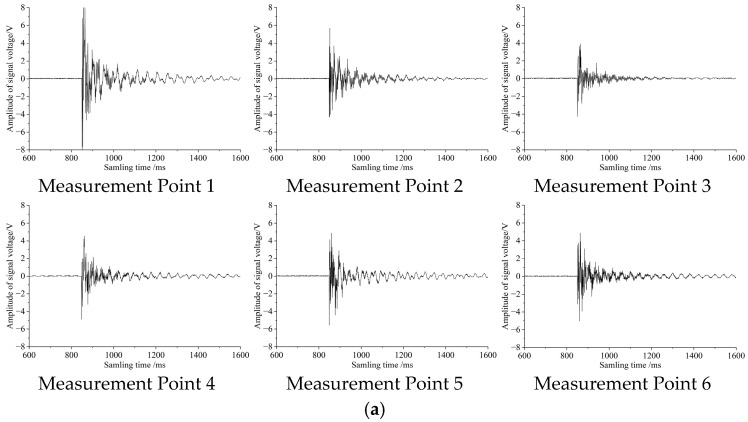

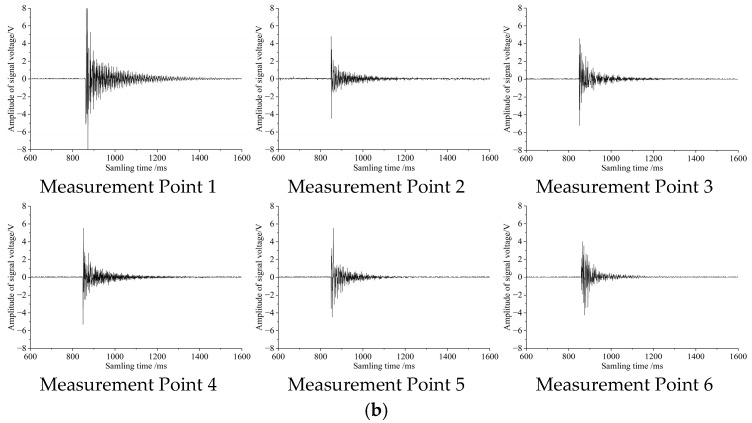
Voltage signal waveforms when grains collide with sensitive plates of different shapes. (**a**) Voltage signal waveforms of circular sensitive plates. (**b**) Voltage signal waveforms of rectangular sensitive plates.

To ultimately select the optimal sensitive plate structure, this study fabricated circular and rectangular sensitive plate specimens using 304 stainless steel and attached piezoelectric ceramic sensors to form detection units. During the experiment, soybean grains of uniform size and mass were released to fall freely from a height of 20 cm above the sensitive plate, impacting different positions as marked in Figure 5—this was to investigate the effect of collision position on the response signal. The output signals, after processing by the conditioning circuit, were recorded using an oscilloscope, and their waveforms are presented in Figure 6.

Analysis of the results in Figure 5a (circular plate) yields the following observations: When the grain impacts the center point (Point 1), the output voltage amplitude (U) is the largest and the decay time (t) is the longest; when impacting the outermost edge point (Point 3), both U and t are the smallest; when impacting intermediate points (Points 2, 4, 5, 6), U and t fall between these two extremes and exhibit numerical similarity. Figure 5b (rectangular plate) shows a comparable trend: the signal response at the center point (Point 1) is the strongest, the response at edge points (Points 3, 5, 6) is the weakest, and the response at the remaining points (Points 2, 4) is intermediate. These results indicate that for both sensitive plate structures, the output signal amplitude (U) and decay time (t) decrease as the distance between the impact point and the piezoelectric sensor increases.

A comparison between Figure 5a (circular plate) and Figure 5b (rectangular plate) shows that the sensing performance of the circular sensitive plate is significantly superior to that of the rectangular one: when grains impact corresponding positions, the circular plate exhibits a noticeably shorter signal decay time (t) and a slightly higher output voltage amplitude (U). This result confirms that the circular sensitive plate possesses both higher response speed—enabling the detection of more grains per unit time—and higher sensing sensitivity. Its comprehensive performance makes it more suitable for high-speed, high-precision grain loss monitoring scenarios. This advantage primarily originates from the higher structural stiffness and more favorable vibration characteristics afforded by its geometric symmetry.

To systematically validate the conclusions from the aforementioned modal analysis and single-point collision test and further explore the quantitative effects of sensitive plate structural parameters on sensor output performance, this study conducted parameterized verification tests. Within the parameter range of thickness (0.5–1.5 mm), rectangular plate width (60–120 mm), and circular plate radius (40–80 mm), the tests comprehensively analyzed the sensor’s response characteristics under various structural parameter combinations.

For each set of parameters, 10 repeated grain collision tests were performed to ensure data reliability. The output signal voltage amplitude (U) was recorded directly, and the average time (t) for the signal to decay to 3.5 V was calculated. The corresponding test results are presented in Table 1 and Table 2.

(1) Regarding signal amplitude (U): At the same thickness, the output voltage amplitude of the circular sensitive plate is consistently higher than that of the rectangular sensitive plate—consistent with the previously obtained conclusion that the circular plate has a larger relative deformation rate. Meanwhile, as the thickness of the sensitive plate increases, the amplitude of both plate shapes shows a downward trend, which is attributed to the increased structural stiffness (stiffer structures restrict deformation, reducing signal amplitude).
(2) Regarding decay time (t): Under all parameter combinations, the signal decay time of the circular sensitive plate is significantly shorter than that of the rectangular sensitive plate. This further verifies that the circular plate’s higher natural frequency directly leads to a faster dynamic response speed, thereby enabling the detection of more grains per unit time.
(3) Confirmation of optimal parameters: Through comprehensive comparison of all parameter combinations, the circular 304 stainless steel sensitive plate with a radius of 60 mm and thickness of 1 mm achieves the best balance between signal amplitude (reflecting sensitivity) and decay time (reflecting response speed). This size not only ensures reliable detection performance but also offers good structural rationality and ease of installation, providing an optimal sensor configuration for grain loss monitoring in combine harvesters.

## 4. Design of Grain Loss Sensor System

### 4.1. Sensor Circuit Structure

**Figure 7:**
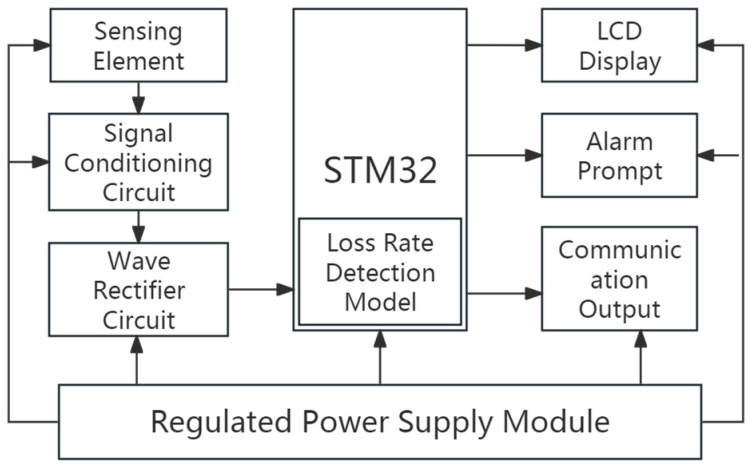
Overall architecture diagram of the system.

A circular 304 stainless steel plate was selected as the sensitive plate for the grain loss rate sensor design [23]. The grain loss monitoring system adopts the STM32F103 microcontroller as its core. During operation, when impacted by falling grains, the piezoelectric sensitive plate generates a charge signal. This signal is converted into a voltage pulse via a signal conditioning circuit, then captured by the microcontroller’s interrupt port for pulse counting. Based on a pre-established grain loss calculation model, the microcontroller computes the real-time loss rate and displays the result on an LCD screen. The overall system architecture is presented in Figure 7.

### 4.2. Design and Performance Optimization of the Signal Conditioning Circuit

The signal conditioning circuit is the core unit for achieving accurate recognition of grains in the system, and it mainly consists of a charge amplifier, a band-pass filter, and a voltage comparator. Among them, the charge amplifier converts the high-impedance charge signals output by the piezoelectric element into low-impedance voltage signals; the band-pass filter is used to extract the grain collision signals in the target frequency band; the voltage comparator distinguishes between effective signals and interference signals through an amplitude threshold.

### 4.3. Design of the Cutoff Frequency for the Band-Pass Filter

To accurately extract the characteristic signals of grain collisions from the complex impact signals of the threshed mixture, a 4th-order Butterworth band-pass filter (composed of one second-order high-pass filter and one second-order low-pass filter connected in series) is adopted. The passband ripple is set to ≤0.5 dB, and the quality factor Q = 1.25.

**Figure 8:**
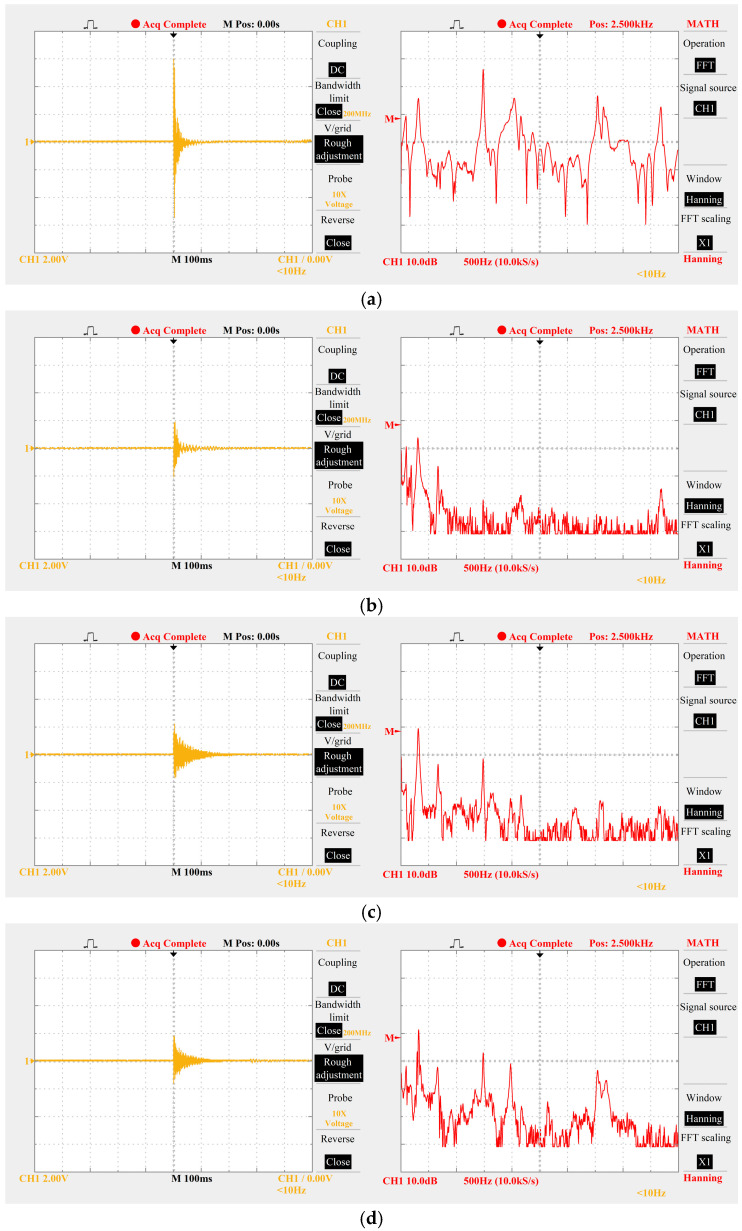
Time-domain and frequency-domain signals generated by different materials striking sensitive plates. (**a**) Time-domain and frequency-domain signals of plump grains. (**b**) Time-domain signals and frequency-domain signals of bean pods. (**c**) Time-domain signals and frequency-domain signals of short stalks. (**d**) time-domain signals and frequency-domain signals of long stalks.

Fast Fourier Transform (FFT) was employed to conduct spectral analysis on the impact signals of different materials (including plump grains, pods, and straw), with the results presented in Figure 8. Extensive experimental results demonstrate the following:

The dominant frequency of impact signals from plump grains is concentrated in the 1.0–3.0 kHz band;

The dominant frequency of impurity signals (e.g., from machine vibration, pods, and straw) is mostly below 500 Hz, and the non-dominant frequency components of impurities in the 1.0–3.0 kHz band are “weak signals” (characterized by low energy proportion and small amplitude).

Based on these findings, the cutoff frequency of the band-pass filter is set to 1.0–3.0 kHz. This setting enables two key functions: on the one hand, it completely filters out strong low-frequency clutter below 500 Hz; on the other hand, it retains the dominant grain signals and weak impurity interference signals within this band, laying a foundation for the subsequent amplitude screening of the voltage comparator.

### 4.4. Determination of the Optimal Threshold for the Voltage Comparator

Under actual field operating conditions, grain signals are far fewer than impurity signals, which constitutes an imbalanced sample scenario. The Precision-Recall (PR) curve is more sensitive to the recognition performance of “a small number of positive samples (grains)”. Thus, the PR curve is employed to quantify the balance between “prevention of false judgment of impurities (Precision)” and “prevention of missed judgment of grains (Recall)”, for the purpose of determining the optimal threshold of the voltage comparator. The specific process is as follows:

(1) Threshold Traversal and Sample Classification: Based on the signals filtered in the 1.0–3.0 kHz band, a series of candidate voltage thresholds were selected. For each threshold, the binary classification of signals into “grain (positive class)” or “impurity (negative class)” was performed, and three key indicators were counted:

True Positive (TP): The number of signals that are actually grains and correctly identified as grains;

False Positive (FP): The number of signals that are actually impurities but incorrectly classified as grains;

False Negative (FN): The number of signals that are actually grains but missed and classified as impurities.

(2) Construction of the PR Curve: For each candidate threshold, Precision and Recall were calculated. Precision is defined as $Precision=\frac{TP}{TP+FP}$, which reflects the ability to suppress false judgments of impurities; Recall is defined as $Recall=\frac{TP}{TP+FN}$, which reflects the ability to suppress missed judgments of grains.

**Figure 9:**
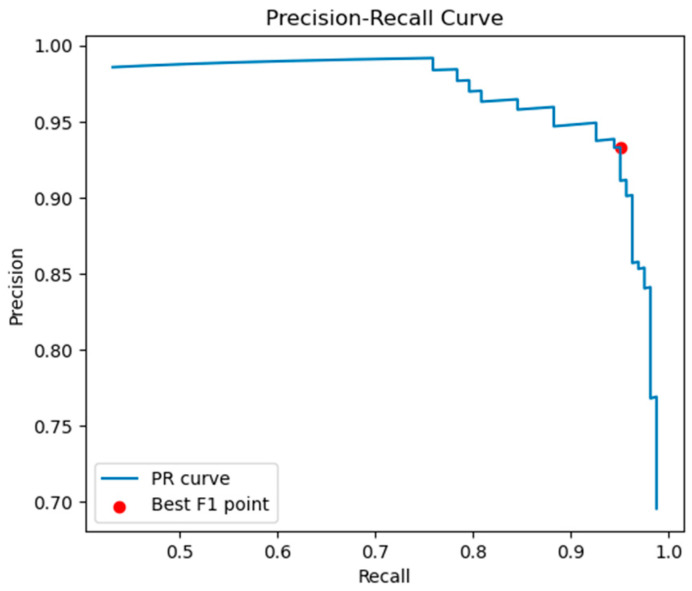
PR curve for optimal threshold selection of voltage comparator based on 1.0–3.0 kHz filtered signals.

The coordinate points of “Recall (x-axis) − Precision (y-axis)” were connected sequentially, forming the PR curve, as shown in Figure 9.

(3) Selection of the Optimal Threshold: The F1-score is the harmonic mean of Precision and Recall, with the formula $F1=\frac{2\times Precision\times Recall}{Precision+Recall}$; it can comprehensively evaluate the balanced performance between “false judgment and missed judgment”.

On the PR curve, the F1-score corresponding to each threshold was calculated, and the point with the highest F1-score was selected as the “optimal balance point of Precision and Recall” (i.e., the red-marked point in Figure 9). This point corresponds to a voltage threshold of 3.5 V. At this threshold, the system achieved a Precision of 93.3% and a Recall of 95%, with the optimal F1-score. Thus, the voltage threshold was determined as 3.5 V.

### 4.5. Digital Circuits and Counting Algorithm Design

The conditioned standard TTL pulse signals are sent to the STM32 (STMicroelectronics, Geneva, Switzerland) microcontroller for counting. To address the counting errors caused by the secondary rebound of grains and the simultaneous collision of multiple grains, this study designed an intelligent counting algorithm based on high-level duration.

**Figure 10:**
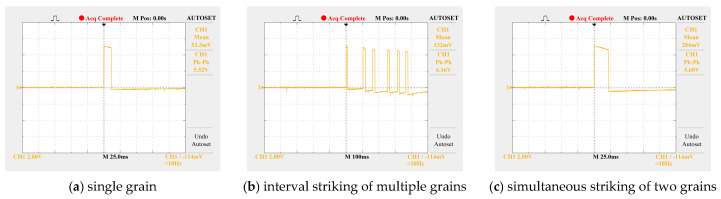
High-level duration of signals when grains strike sensitive plates.

Tests show that the average width of the high-level pulse generated by the collision of a single grain is approximately 10 ms. When multiple grains collide simultaneously, the pulse width increases significantly. The algorithm distinguishes between single-grain and multi-grain collision events by judging the duration of the high-level signal through software (for example: a duration within 10 ms is counted as 1 grain, a duration between 10 and 20 ms is counted as 2 grains, and so on). Field tests indicate that in most cases, the number of grains colliding simultaneously does not exceed 3, and this algorithm can effectively improve counting accuracy, as shown in Figure 10. When grains collide with the sensor and cause secondary rebound, the electrical signals generated by the rebound can be filtered out by the set voltage comparator.

## 5. Performance Test of Grain Loss Sensor

### 5.1. Laboratory Calibration Test

**Figure 11:**
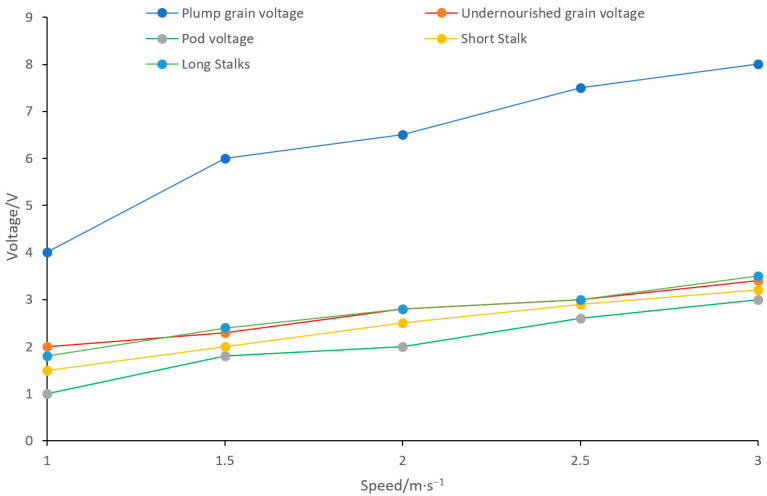
curves of output voltage peak variation for different materials at different impact velocities.

To determine the optimal threshold voltage of the voltage comparator, a dedicated experiment was conducted under laboratory conditions: different materials—including plump grains, shrunken grains, pods, and straw—were ejected at a controlled speed range of 1–3 m/s to impact the sensitive plate (consistent with the circular 304 stainless steel sensitive plate used earlier). As shown in Figure 11, the results indicate that the peak output voltage of plump grains increases with collision speed, ranging from 3.5 V to 8.0 V. In contrast, the peak voltages of other impurity materials (shrunken grains, pods, straw) are all below 3.5 V. This clear distinction in peak voltage enables the following optimization: setting the voltage comparator’s threshold voltage to 3.5 V can maximize the accurate detection of plump grains while effectively suppressing interference signals from impurities.

### 5.2. Detection of Sensor Recognition Ability Under Mixed Materials

Under laboratory conditions with a grain drop height of 200 mm and a sensor-sensitive plate installation angle of 30°, a recognition test was conducted on mixed materials (plump soybean grains, shrunken grains, pods, and straw). Each group of calibration tests was repeated 3 times, and the average value was calculated to ensure data reliability. As shown in Table 3, the sensor’s recognition accuracy for plump soybean grains reached up to 95.1%. This result confirms its excellent detection precision and anti-interference capability, which is consistent with the threshold optimization results (Section 4.4) that 3.5 V effectively suppresses impurity interference.

### 5.3. Field Tests

**Figure 12:**
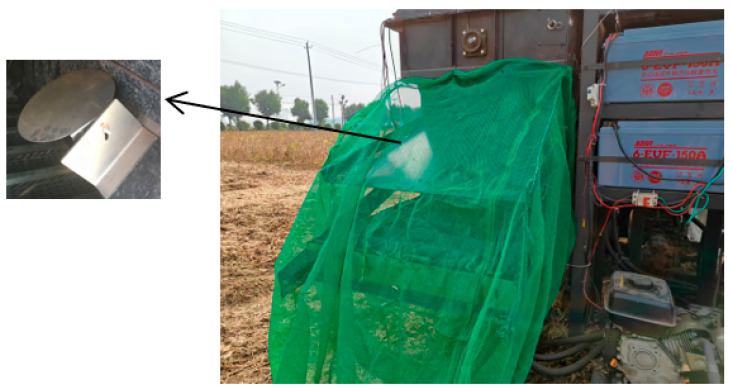
Field experiment diagram.

In October 2024, the developed grain loss monitoring sensor was installed on a combine harvester, and a field soybean harvesting test was conducted in Xianggong Town, Linyi City, Shandong Province. The test process is illustrated in Figure 12.

Before the test, the threshold voltage was set to 3.5 V. When the machine was operating under no-load conditions, the system count remained consistently at 0. This indicated that the vibration of the harvester itself and ground jolts did not generate valid signals, demonstrating the system’s excellent anti-interference performance.

During the formal tests, a proportional coefficient K—which correlates the system’s count value with the actual number of lost grains—was first calibrated by comparing manual collection results with the system’s count data. After implanting this coefficient K into the system, five tests were conducted, each under different operating parameters. As shown in Table 4, the loss rate measured by the system was highly consistent with the manually measured values (with an average relative error of <4%). This fully verifies the reliability and accuracy of the grain loss sensor system developed in this study during actual operations.

## 6. Conclusions

Centering on the core goal of improving the grain loss monitoring performance of combine harvesters, this study draws the following conclusions through theoretical analysis, simulation optimization, and experimental verification:

(1) The vibration characteristics of the sensitive plate determine the sensor performance. Theoretical analysis and simulations show that the first-order natural frequency dominates the signal decay rate, while the relative deformation rate determines the signal amplitude.
(2) The circular 304 stainless steel sensitive plate exhibits significant advantages in comprehensive performance due to its unique structural characteristics. Compared with the rectangular plate, it has a higher natural frequency and a shorter signal attenuation time while maintaining a relatively high output sensitivity.
(3) The signal processing strategy based on FFT (Fast Fourier Transform) spectrum analysis (1.0–3.0 kHz band-pass filtering) and a 3.5 V voltage threshold can effectively distinguish between grain and impurity signals. Combined with the counting algorithm based on high-level duration, this strategy solves the counting error problems caused by multi-grain collision and secondary rebound.
(4) Field tests indicate that the developed sensor system has strong anti-interference capability, with a measurement error of less than 4%, which meets the requirements of actual operations and provides an effective technical means for reducing grain harvesting losses.

This study provides an important theoretical basis and practical solutions for the development of high-performance grain loss monitoring sensors.

## 7. Future Research

At the current stage, the sensor’s anti-interference capability has been preliminarily verified under laboratory-controlled conditions and simple field scenarios, though comprehensive validation under complex actual operating conditions—encompassing vibration, fluctuations in feed rate, temperature and humidity variations, and coupled dust-moisture environments—and in-depth elaboration of the signal processing algorithm remain incomplete. These aspects will serve as the core focus of future research.

## Figures and Tables

**Table 1:** Voltage signal amplitude and time to decay to 3.5 V for rectangular sensitive plates of different sizes.

Thickness/mm	Width 60 mm		Width 90 mm		Width 120 mm	
	Amplitude/V	Time/ms	Amplitude/V	Time/ms	Amplitude/V	Time/ms
0.5	7.3–8.0	12.0–15.0	6.8–7.5	15.0–18.0	6.3–7.2	15.0–20.0
1.0	6.5–7.5	8.0–12.0	6.5–7.5	8.0–12.0	6.2–7.0	9.0–12.0
1.5	4.5–6.0	7.0–10.0	4.5–5.8	8.0–10.0	4.5–5.5	8.5–10.0

**Table 2:** Voltage signal amplitude and time to decay to 3.5 V for circular sensitive plates of different sizes.

Thickness/mm	Radius 40 mm		Radius 60 mm		Radius 80 mm	
	Amplitude/V	Time/ms	Amplitude/V	Time/ms	Amplitude/V	Time/ms
0.5	7.5–8.0	10.0–12.0	7.5–8.0	10.0–15.0	7.5–8.0	10.0–15.0
1.0	6.5–7.8	5.5–10.0	7.0–8.0	6.5–10.0	7.2–8.0	7.0–10.0
1.5	5.0–6.5	5.0–8.0	5.5–7.0	5.5–8.0	5.5–7.5	6.0–8.0

**Table 3:** Detection error of grain loss monitoring sensors under different material conditions.

Collision Material Quantity	Monitoring Quantity per Grain	Relative Error/%
100 Undeveloped Grains	2	2
Bean pod	0	
Short Stalks	0	
Long Stalks	0	
1000 Plump Grains, 100 Undeveloped Grains, Bean Pods, Short Stalks, Long Stalks	1049	4.9

**Table 4:** Comparison between sensor data and manual data in field experiments.

Test Number	Cleaning Loss				Absolute Error/%	Relative Error/%
	Sensor Detection		Manual Detection			
	Number of Lost Grains (Grains)	Loss Rate (%)	Number of Lost Grains (Grains)	Loss Rate (%)		
1	100	0.55	105	0.58	0.03	3.03
2	90	0.50	93	0.52	0.02	3.84
3	98	0.54	102	0.56	0.02	3.57
4	134	0.74	129	0.72	0.02	2.77
5	124	0.68	119	0.66	0.02	3.03

Note: The 100-seed weight of soybeans is 25 g, the yield per unit area is 0.225 kg, the cutting width of the harvester is 2 m, and the harvesting length is 10 m.

## Data Availability

Data are contained within the article.
